# The Political Economy of Longevity: Developing New Forms of Solidarity for Later Life

**DOI:** 10.1111/tsq.12082

**Published:** 2015-01-09

**Authors:** Chris Phillipson

**Affiliations:** The University of Manchester

## Abstract

Aging populations now exert influence on all aspects of social life. This article examines changes to major social and economic institutions linked with old age, taking the period from the mid-20th century to the opening decades of the 21st century. These developments are set within the context of the influence of globalization as well as the impact of the 2008 financial crisis, these restructuring debates around the longevity revolution. The article examines how the basis for a new framework for accommodating longevity can be built, outlining ways of securing new forms of solidarity in later life.

## Introduction

Aging populations now exert a major influence on all aspects of social and economic life. Concerns about the most appropriate way of resourcing such populations, their impact on standards of living, and relations between age groups and generations feature prominently in public debate and discussion. The 21st century will without question be a time when all societies take stock of the long-term impact of demographic change and the implications for managing and organizing a major area of social and economic activity. Thus far, discussions have been tentative at best, discriminatory at worst, focusing on the apparent “cost” and “burden” associated with population aging. Doubts about the value and purpose of longevity seem, if anything, to have become more not less strident in the present century (Baars et al. 2013).

Anxieties about the challenges arising from longevity reflect a number of issues. First, the pace of population change is one important dimension (Victor 2010). Demographic projections indicate that many societies across the globe can anticipate having up to one-third or more of their populations aged 60 years and over by 2050, with substantial increases in many cases. China, for example, is likely to reach around half a billion people aged 60 years and over by the middle of the 21st century (Yi and George 2010). Second, the “intensification” of aging—resulting from the continuing falls in fertility and continuing gains in longevity—has coincided with a crisis in confidence affecting institutions arising from the financial crash of 2008 and subsequent economic recession. In this context, population aging—often viewed as a “mixed blessing” by Western governments—has assumed even more negative connotations. Demographic change could not be said to have “caused” the economic crisis, but—it is argued—finding solutions will become ever more difficult given the “rising tide” of elderly people and the health and social problems that they bring (Liedtke and Schanz 2012). Increasingly, governments point to population aging as a way of explaining why resolving economic problems will be difficult and why the pain incurred in searching for solutions will create concerns for all age groups. Third, aging has emerged as a “problem” (at least in Western societies) because of the challenges to the welfare state, with increased emphasis on private (individual) rather than public (collective) provision (Judt 2010).

The aim of this article is to examine some of the sociological implications of population aging in the 21st century. The article first examines changes to major social and economic institutions linked with old age, taking the period from the mid-20th century to the opening decades of the 21st century. Second, these developments will be set within the context of the influence of globalization as well as the impact of the 2008 financial crisis, both of these viewed as destabilizing institutional supports for aging and restructuring debates around the longevity revolution. Third, the article will examine how the basis for a new framework for accommodating longevity can be built, contrasting perspectives around the idea of the “third age” with those concerned with securing new forms of solidarity in later life.

## Modernizing Old Age

A starting point for the discussion is the way in which old age was transformed in the two decades following the ending of World War II. The key development here concerns how, in advanced capitalist countries, growing old was reconstructed through the social and economic institutions linked with mandatory retirement and the welfare state. These became crucial in shaping the dominant discourse around which aging was framed, and the identities with which it was associated. A supporting theme was the reordering of the life course into distinctive stages (or “boxes” [Best 1980; Phillipson 2002] as they came to be termed) associated with education, work, and retirement, with the transition between the last two becoming an important element in the fashioning of a new identity separate from that associated with work and paid employment (Grenier 2012).

Older people—as a social category—can be viewed as a creation of modernity, reflecting the achievements of industrialization, improved public health, and the growth of social welfare (Achenbaum 2010). From the 1950s to the 1970s, the umbrella provided by retirement and the welfare state appeared to have created a degree of security for old age (Phillipson 1998). The meaning of later life was, temporarily at least, developed through a vision where retirement and welfare were viewed as natural supports to the end of the human life cycle. The idea of “retirement” was an essential part of the narrative, driving the reconstruction of aging (Blaikie 1999). In the 1950s and 1960s, retirement at age 60 (in the case of women) and 65 (in the case of men) years became widely established. Indeed, “By the late 1960s, it was accepted that the *normal* period of full-time employment would cease for *most* of the population at these ages” (Harper and Thane 1989:59). Retirement thus became an important “social institution,” regulating the passage from work to the final stages of the life course (Kohli 1986).

From a sociological perspective, two contrasting features emerge in the late 1960s through to the 1980s regarding the experience of work in middle and later life. First, leaving employment came to be viewed (especially for men) as part of a “mass transition,” with associated “rites of passage” (Crawford 1971). Linked to this was an emphasis on the value of retirement as a “distinctive” phase in life, together with the importance of “preparation” for the event (Help the Aged 1979). Building on this came the idea of a new stage in the life course—the “third age”—advanced by Laslett (1989; see further below), which identified new opportunities for personal fulfillment following the ending of full-time work.

However, almost as soon as the outlines of a settled period had appeared, it became clear that changes affecting working life were beginning to undermine the institution of retirement. Researchers drew a distinction between “retirement” on one side and “early exit” on the other side (Kohli et al. 1991). The former referred to reaching the state's definition of retirement for state pension purposes; the latter, the point at which older workers made an early withdrawal—using early retirement, unemployment, disability, or associated benefits—from paid employment. Rather than employment ending after a set number of years and at a fixed chronological age, there was now a measure of ambiguity about the ending of work and the beginning of retirement (Schuller 1989). Kohli (1986) and Guillemard (1989) argued that evidence that people were withdrawing from work at earlier ages suggested the “breakup” of the type of retirement that had emerged across most Western industrial countries in the 1950s and 1960s. Guillemard (1989) commented that there was

less and less of a definite order to the last phase in life. The life course is being de-institutionalized. Along with the abandonment of conventional retirement, we also see the break-up of … the threefold model which placed the individual in a foreseeable life course of continuous, consecutive sequences of functions and statuses. As a consequence, an individual's working life now ends in confusion. (P. 177)

Such views indicated a new form of crisis in the lives of older people, reflecting the restructuring of work in the 1970s and 1980s alongside pressures on the welfare state (Phillipson 1998). Work became less secure during this period, with the rise of “flexible” employment, short-term contracts, and the downsizing of industries (Sennett 1998). For older workers, the significance of economic decline was reinforced by a demographic context where a substantial cohort of younger people was now entering the labor market. Older workers became targeted as a key group to remove from employment, reflected in the terminology of the period, that is, “redundancies,” “shakeouts,” and “early retirement” (Laczko and Phillipson 1991).

Support for retirement as a major stage in the life course came under pressure from the mid-1990s, with debates moving back to emphasizing the importance of older people retaining an attachment to work and the “unaffordable” nature of retirement in its traditional form (Liedtke and Schanz 2012). Governments across most industrial countries became concerned about the economic consequences of aging populations and associated costs of pensions and care services (OECD 2006). The age at which people could gain access to state pensions was raised in many countries alongside the introduction of measures to tackle age discrimination in the workplace.

At the same time, it is clear that the swing back to encourage older people to remain in employment raised a number of problems. Ambiguity remains in terms of the management of “work-ending.” Indeed, in countries such as the United Kingdom (UK), a virtual majority of men and women still leave employment ahead of their 65th birthday (one-quarter of men are out of the workforce before age 59 years). The evidence from countries such as the UK and United States is that once out of employment, those who are 50 years old and over find it harder to get back into work in comparison with young people (Ekerdt 2010; Cory 2012). Raising pension and social security ages are likely to be especially detrimental to low-income and minority workers, these groups suffering from poorer health and lower life expectancy in comparison with high-income groups (Angel and Mudrazija 2011). The impact of large-scale unemployment, in high- as well as low-income countries, has in any event created restrictions on the opportunities available to older employees, with many joining the ranks of those experiencing chronic insecurity in the labor market—the “precariat” as defined by Standing (2011). Reflecting these changes, and even before the economic crisis of 2008, researchers had begun to identify retirement as involving new risks and insecurities. On the one side, retirement appeared to have been “reinvented as a time of transition to a new life, rather than simply an old one” (Hockey and James 2003:102). On the other hand, this “new life” began to take on many trappings of the old one in respect of a deepening of social and economic inequalities (Vickerstaff and Cox 2005).

## Changing Constructions of Old Age

From the 1990s onward, the crisis affecting retirement and the welfare state illustrated the way social marginality among the old had been contained rather than resolved. Moreover, what a postindustrial society did have to offer—the shift in emphasis from production to consumption—seemed only to create further difficulties for those living on the economic margins or displaced from employment. Bauman (1992), for example, writes of the accelerating emancipation of capital from labor, producing a situation where “instead of engaging the rest of society in the role of producers, capital tends to engage them in the role of consumers” (Bauman 1992:111; see also Gilleard and Higgs 2005). This development was to challenge definitions of what it meant to be an “older person” and associated ideologies of support. Growing old moved from a focus on collective responsibilities toward an emphasis on the way in which families and individuals should themselves handle the demands associated with population aging (see further below).

Improved life expectancy may itself be viewed as a consequence of the social transformations associated with modernization. Beck (1992:21, author's emphasis) defines the nature of risk as a “*systematic way of dealing with hazards and insecurities induced and introduced by modernization itself*.” Of course, older people have been ever present in human history, and often in positions emphasizing their relative powerlessness faced with poverty, bereavement, and ill health. But to paraphrase Beck, in the past these could be seen as personal rather than societal tragedies. The impact of older people was restricted by the relatively superficial nature of the category of “pensioner” or “elderly person” and by their limited demographic profile. Changes accompanying modernization have transformed both these elements, with aging becoming a socially recognized risk, but with a gradual weakening in the institutional supports associated with the “social” or “welfare” state (Piketty 2014).

However, three additional characteristics have become apparent with the maturation of aging populations. First, the “global” dimensions of aging are increasingly important (Higo and Williamson 2011). All societies (poor as well as rich) are experiencing growth both in the numbers and proportions of older people in their populations (Victor 2010). Aging thus becomes simultaneously both a biographical event and one shared with different cultures and societies across the globe. Second, aging populations assume increasing diversity in respect of incomes and lifestyles, reflecting contrasting experiences between and within birth cohorts (Formosa and Higgs 2013). Third, older people transform, as well as being transformed by, social and economic institutions. Beck (1992:10) captures this dimension, where he argues that “the more societies are modernized the more agents (subjects) acquire the ability to reflect upon the social conditions of their existence and to change them in that way.” For older people, this raised the possibility of moving from conditions of dependency toward embracing consumer-based lifestyles (Gilleard and Higgs 2005). Against this, Vickerstaff and Cox (2005:92) suggest that the result of what they term the “individualization of retirement” has been “less to increase the majority of people's range of alternatives and choices over when and how to retire and more to enlarge the range of risks they [have] to cope with.” The weakening in supports underpinning longevity have, however, been exacerbated by the wider structural and ideological processes associated with globalization, a summary of which is presented below.

## Globalization and Risk

Debates around the impact of globalization on aging have been extensive both within the social gerontology literature (see, for example, Baars et al. 2006; Dannefer and Phillipson 2010) and in studies of social policy (Yeates 2008). One argument emerging from within gerontology concerns the extent to which globalization has itself become an influential factor in the construction of old age, notably in the design of policies aimed at regulating and managing population aging. Although the impact of globalization remains “highly contested” (Diamond 2010), an interdependent world such as that associated with more fluid labor markets and transnational forms of governance appears to create distinctive pressures across the life course. Yeates (2001:2) suggests that the relationship between globalization and social policy is best conceived as “dialectical” or “reciprocal” and that “far from states, welfare states and populations passively ‘receiving’ [and] adapting to globalization…they are active participants in its development” (see, further, Diamond 2010). This may be especially the case in the context of the post-2008 recession, where nation states appear to have played an enhanced role in the development of economic and social policies (Gray 2010).

The processes associated with globalization have assisted the development of a new approach to aging societies, based around what Ferge (1997) refers to as the “individualization of the social.” On the one side, aging is presented as a global problem and concern; on the other side, the focus has moved toward individualizing the various risks attached to growing old. Young (1999:6) interpreted such developments as part of a wider shift from an “*inclusive* to an *exclusive* society. That is from a society whose accent was on assimilation and incorporation to one that separates and excludes. This erosion of the inclusive world … involved processes of disaggregation both in the sphere of community (the rise of individualism) and the sphere of work (transformation of … labor markets). Both processes are the result of market forces and their transformation by the human actors involved” (see also Scharf and Keating 2012). From a sociological perspective, writers such as Bauman (1998) presented the “human consequences” of globalization in terms of new forms of segregation and exploitation, especially affecting those in deprived and peripheral communities (see Sassen 2014). And Sennett (2006) linked aspects of globalization—for example, the rise of flexible labor markets—to the “erosion of social capitalism,” with older workers increasingly disadvantaged within corporations that emphasize low-wage and low-skill work environments (Standing 2011).

All of the above carries significant implications for understanding the landscape influencing longevity over the next phase of its development. The impact of globalization, alongside and interacting with a transformed life course, has redefined the social context of aging. Population aging has now to be “managed” within what has been described as a fluid and deregulated social order (Elliott and Lemert 2006), thus opening the possibility for inequalities within periods such as later life to find new forms of expression. Risks once carried by social institutions have now been displaced onto the shoulders of individuals and/or their families. Dannefer (2000:270) summarizes this process in the following way: “Corporate and state uncertainties are transferred to citizens—protecting large institutions while exposing individuals to a possible catastrophe in the domains of health care and personal finances, justified to the public by the claim that the pensioner can do better on his or own… .” At the same time, the evidence suggests widening inequalities within and between different countries, produced as a consequence of global forces. Rather than leading inexorably to minimum levels of social protection, globalization has been implicated in the rise in income inequality produced as a consequence of falling relative demand for unskilled labor and the weakened power of labor organizations (Therborn 2013). The rapid increase in incomes at the very top of the income distribution has been a feature of advanced industrial societies throughout the 1990s and 2000s (Piketty 2014). For those less fortunate, however, there has been the growth of what Sennett (2006) refers to as “underemployment,” this coming alongside restrictions on the growth of wages and salaries, both coming with greater insecurity in the labor market accompanying economic globalization (Clark 2014).

## Reconstructing Aging: Changing Institutions and Generations

Periods of economic recession and growth alongside the impact of globalization, have produced greater cultural and social diversity (and inequality) to aging. The shift here is from the “stages of life” characteristic of pre- and early modern times, toward a more fluid and unstable landscape surrounding the latter end of the life course. Polivka (2000) views this development as reflecting the increasingly “improvisational” nature of the life course, with people cultivating the capacity to adjust to discontinuity in key areas of their life. Such changes raise questions about the way in which social aging is evolving, given the restructuring of retirement and the welfare state. Settersten and Trauten (2009) suggest that for older people, the future seems open, but also fragile. They observe that

Choices now seem greater, but these choices seem heavier and come with unknown consequences. Any fallouts must be negotiated and absorbed by individuals and their families rather than by governments, markets, or other entities … the trend towards individualization means that old people are increasingly left to their own devices to determine the directions that the ends of their lives will take. Old people are largely on their own with only the safety nets they can create with the resources they have, whether through personal and family resources or through social skills and psychological capacities. … (Pp. 457–8)

Mandatory retirement and the welfare state—despite their limitations—did begin to provide a supportive framework around which aging could be built. The uncertainty affecting both institutions substantially deepened over the course of the 1990s and 2000s (Baars et al. 2006; Estes and Associates 2001). Older people were enjoined to work even though the possibilities for employment appeared increasingly limited; they were tasked with creating their own sources of income over and above that provided by the state, even while personal pensions declined in value; they were faced with organizing their own social care, under the guise of “personalization,” even though standards and quality of support were placed increasingly in doubt (Phillipson 2013).

The unraveling of retirement and the welfare state has exposed cultural uncertainties surrounding old age (Cole 1992; Baars et al. 2013). Particular groups, such as the baby boom generation, have been singled out as causing particular problems—creating tensions between generations and drawing down a disproportionate share of economic resources. Willetts (2010) summarizes the issues as follows:

At the moment this [baby boom] generation dominates just about every important institution in the country: it has most of the wealth and power. How will this generation discharge its obligations to younger generations? So far it has been one of the luckiest generations. Will the boomers be selfish with their luck, or will they pass it on to the next generation? So far the evidence is not good. The baby boomers, having enjoyed so far a spectacularly good deal, are dumping too many problems on the younger generation. It has the great advantage of being a giant generation but how will it use that power? At the moment it looks like a selfish giant. (P. xxi)

There are, however, a number of problems with this type of argument: the UK baby boom could never be described as a “giant generation” in the way that might in some respects be accurate for countries such as Australia and the United States. Unlike the latter two, the UK did not experience a sustained baby boom from the mid-1940s through to the mid-1960s. Rather, there were two separate “spikes”—in the late 1940s to the early 1950s (best termed—as it was at the time—a “bulge” rather than a “boom”)—and then another in the early 1960s. Birth cohorts, however, whatever their size tend not to behave either in “selfish” or “altruistic” ways; instead, political action is generally fragmented by the usual markers of class, gender, and ethnicity (Abrams 1982). And certainly not all baby boomers have enjoyed a “spectacularly good deal,” especially in terms of access to resources for old age. Portes (2014:F9) in a review of data on generational equity concludes that “the simple story of coddled pensioners and struggling youth does not really add up: both inequality and redistribution within generations remains much greater than between generations—and its impact is growing.”

But the accuracy of arguments about baby boomers is probably less important than the view that a particular group is about to reap “undeserved” benefits in old age. Underlying this appears to be a suggestion that support provided by the welfare state is likely to switch from the poor and the young to the old and the rich. Concerns about the costs of support for old people are not themselves new. They had a relatively low profile in the 1950s and 1960s in the context of full employment and the building of the welfare state (Kynaston 2009). They surfaced in the late 1970s in the context of economic crisis and the rise of neoliberal governments, with emphasis placed on the pressure on working family budgets from pension contributions and related benefits (Longman 1987). And they returned again in the 2000s, when a combination of the economic recession and boomers moving into retirement led to concerns that older people were gaining economic benefits at the expense of the young (Howker and Malik 2010; Willetts 2010). These shifts in perceptions about aging populations indicate fragility in the construction of aging, but in the context of changing approaches to managing an extended life course. A particular feature here is the emphasis on personal responsibility for health in later life and the distinction between the “third” and “fourth” age. The next section reviews these areas in terms of representing a distinctive stage in the development of ideas about aging.

## Longevity without Modernity

The argument that follows from the preceding sections is that aging in the 21st century has to be managed stripped of the institutions, which evolved over the 20th century—and especially those which emerged from the 1940s. This loosening of aging from the institutions of modernity has arrived at precisely the point at which the possibilities for growing old are being expanded by the various (and competing) claims for health enhancement and extending longevity (see Dumas and Turner 2015; also Vincent 2010). Moreover, this process is itself reinforced by a dominant narrative of personal responsibility for health and well-being, set within the context of individualized consumer-based lifestyles (Higgs and Gilleard 2014). The new context for growing old has opened up a range of social and cultural spaces, which are changing the construction of old age. One illustration can be found in the debate about the emergence of the third and fourth ages; another can be found in the possibilities of new forms of solidarity for later life. Both these areas will now be discussed and their implications highlighted for debates around the social context of aging and longevity. The following section discusses this issue as reflected through the distinction between the “third” and “fourth” age.

### The Arrival of the Third Age

The idea of the “third age” was introduced by Laslett (1989) who argued that it was now possible to identify a new stage in life in between that of work (the “second age”) and that of late old age (the “fourth age”). Rather than viewing this period as a residual phase in life, instead it could be seen as offering new possibilities for individual growth and development. Gilleard and Higgs (2000, 2005) elaborated on the sociological bases of the third age by grounding it in the life histories of the baby boom generation “where the dispositions created by the youth culture of the 1960s have continued into retirement and where the generational emphasis on choice and freedom has reset expectations.” Jones et al. (2008) concluded that

it is possible to consider the third age in late modernity as a cultural field where later life becomes more diverse and heterogeneous. Within the context of a post-scarcity westernised life course, it is no longer appropriate to categorise people entering later life as a homogenous group of “retirees,” “third agers” or indeed “pensioners.” Instead post-working life is based around a variety of lifestyles, uncertainties, anxieties and aspirations. Furthermore, the cultural field that comprises the “third age” transcends social distinctions based around age, class, status, race or gender. The crucial underpinning to this cultural field is the rise of mass consumer society … [and] the transformation of consumption practices that successive generations take with them into later life. (P. 114)

This focus on consumption and the third age indicates a cultural space for an antiaging rhetoric, highlighting the possibility for people to shape their own “aging” in a way that was inconceivable for previous generations. According to this argument, people currently reaching retirement age have indeed been “exposed to and [have] participated in an expanding consumer market” (Jones et al. 2008:25) and might as a consequence become assimilated into the commercial drive to promote healthy aging. But two significant problems arise from framing the issues in this way: first, from a political economy perspective, important distributional issues are raised in respect of inequalities within the third age; second, the problem of managing a new “residual” category—that of the “fourth age.”

The first point goes back to an argument made in the previous discussion on the politics of the boomer generation, namely, the fact that “third agers” will almost certainly be heavily stratified by class, gender, and ethnicity. Indeed, if the evidence assembled by Portes (2014) and Piketty (2014) is correct, those heading into their 60s and beyond may show greater socioeconomic divisions as compared with retirees in the period up to the end of the 1970s (see Formosa and Higgs 2013). Some of the relevant factors here include the return to low rates of growth and relative stagnation in wages and salaries (as compared with the period from the 1950s to the 1970s); the move to defined benefit to defined contribution pension schemes, the latter producing uncertainty regarding retirement incomes (Russell 2014); and the inequalities arising from the raising of state pension ages, with unequal access to secure employment (Angel and Mudrazija 2011). The likelihood is that these—and linked factors (such as the weakening in the postwar welfare or social state)—will create major differences within the population defined as belonging to the third age (Higgs and Formosa 2013; Lopas 2013). The importance of this for our purposes is that participation in the activities associated with antiaging will become even more stratified than they are at present, with the likelihood of a “socially excluded” group of older people with limited access to commercialized health enhancement but lacking also access to good quality public care (Harari 2014).

### The “Problem” of the Fourth Age

The second issue highlighted above concerns the problem created by the distinction between the third and fourth age, or the distinction between “healthier” groups of older people and those more prone to impairment and decline. The articulation of the third and fourth age has been criticized for overstating the potential of the third age, defining illness and impairment as negative, and for pushing the stigmatizing aspects of aging into the fourth age (Grenier 2007). In recent years, researchers in gerontology have reappraised frailty and the fourth age in a variety of ways (Grenier 2007; Gilleard and Higgs 2010, 2011). In biomedical studies and geriatrics, the constructs of frailty and the fourth age are used to refer to a diagnosis or medical condition and as a means of targeting resources for health and social care (Rockwood and Mitniski 2007). In the social sciences, frailty and the fourth age has been considered as sociocultural constructs, examples of practices used to classify and determine eligibility for public health and social care services, and symbolic and meaningful locations in late life (see Gilleard and Higgs 2010; Grenier 2012).

Grenier (2007) drew attention to the importance of the symbolic associations of decline that are made with regards to “frail” older bodies in health and social care practices relating to risk and frailty, and later, to the ways in which these beliefs, when considered in the broader sociocultural context, resulted in a growing polarization between the “third” and “fourth age” (see Grenier 2012). Higgs and Gilleard (2014:15) make the point that “for most people most of the time aging can be managed more or less successfully by people distancing themselves from what is perceived as the most oppressive aspects of agedness. For most people, age and aging are not made routinely salient. …” In contrast, in the fourth age, “old age is represented less as a status and more as a state of being, one that is typically envisioned through discourses about the costliness, the frailty and the indignities of old age. Such discursive ‘othering’ … excludes the aged person from the everyday life of society and casts him or her into a position defined equally by its alienation and vulnerability” (Higgs and Gilleard 2014:10).

The way in which the idea of “frailty” is constructed within health systems and society more generally can be viewed as a direct consequence of the emphasis on “healthy life expectancy” or the goal of increasing the number of “illness-free” years. This is the central goal or aspiration of much work in gerontological and related disciplines (Vincent 2010). This is itself reinforced (if not caused) by a wider political project seeking to eradicate the “diseases” of aging (notably those coming under the heading of “dementia”), which are viewed as presenting obstacles to achieving active and successful aging. But the human costs of this project are considerable (Whitehouse 2008). Those consigned to the status of “already frail” become a marginalized group in society, for whom problems of abuse and neglect—in public and private settings—become an everyday threat (Phillipson 1998, 2013). In creating an elevated status for those seeking to hold back the changes associated with aging, those who have “succumbed” to late old age find themselves a low priority for health and social care.

Given the above, looking at “frailty” *differently* has become an urgent issue for a sociology of aging. One important issue here concerns the question of how to conceptualize agency in the context of late old age, as compared with people defined as being in the third age (Tulle 2004). Grenier and Phillipson (2013:72) suggest that agency may *look different* in later life than currently presented. This viewpoint—that the forms or expressions of agency from within the “fourth age” may differ from those which we currently know and expect of agency at other periods of the life course—is a serious challenge for the social sciences. The agency of someone who is in the fourth age, possibly bed-ridden and ill, is likely to be very different from an able-bodied young person. Acts may be nonverbal or take place through forms of communication that are often difficult to understand (for example, cries, moans, or screams) (Bourbonnais and Ducharme 2010). They may also take the form of outright resistance or disruptive acts. What might be argued, however, is that agency from locations such as the “fourth age” may not be characterized by a physically active and public act of collective social change, as is implied in dominant definitions. Arising from this, models are required that integrate more passive and/or less active notions of agency, that take account of constraints ranging from the structural to the personal, and which challenge the underlying assumptions of health, control and independence that are increasingly promoted in current perspectives about growing old (see further Shura, Siders, and Dannefer 2011).

## Creating Solidarity for Aging Populations

The argument developed in this article is that the framework for accommodating aging populations has been fundamentally changed in the 21st century. For a relatively brief period in the previous century, it appeared that longevity could be managed through liberal social policies, economic growth, and a mixture of social and generational ties. An underlying theme, however, concerned the continued responsibility of the state—at least in the European context—in providing support to older people. Old age was largely taken for granted as a time in which public institutions would play a dominant role in the provision of care; a period of life in which it was governments—whether at central or local level—who had primary accountability for ensuring the security and quality of life for the most vulnerable in society. In contrast, the latest period in the development of old age appears underpinned by a new “moral narrative,” to use the phrase of Judt (2010), one represented by a shift from the state to the market, or from aging managed within “public” spaces to within “private” ones (Meyer and Herd 2012). This new phase is by definition more open and less constrained by the traditional institutions supporting older people; equally, it is a more unequal old age—with new opportunities for inclusion on the one side, but varieties of exclusion on the other (Scharf and Keating 2012).

What kind of responses need to be made to these developments: what options do we have for achieving a more secure old age given the economic and social transformations highlighted in this article? A starting point must come from some kind of rethinking of the role of the state in supporting older people. Here, it is important to challenge the idea that the state can progressively withdraw from being a major partner in supporting later life. Of course, the state has not “withered away” in key areas of responsibility. Judt (2010:199) makes the point that “Having reduced the scale of public ownership and intervention over the course of the past thirty years, we now find ourselves embracing *de facto* state action on a scale last seen since the Depression. The reaction against unrestrained financial markets—and the grotesquely disproportionate gains of a few contrasted with the losses of so many—has obliged the state to step in everywhere.” But as Judt also argues, the outstanding point remains that having spent much of the 1990s and 2000s rejecting state/public intervention, the case for why it is needed must be rethought: “We have freed ourselves of the mid-20th century assumption—never universal but certainly widespread—that the state is likely to be *best* solution to any given problem. We now need to liberate ourselves from the opposite notion: that the state is—by definition and always—the *worst* available option” (Judt 2010:202).

But in redefining the role of the state, the question of securing new forms of solidarity across generations and different age/social groups also needs to be considered. Institutions of solidarity have, to use Rosanvallon's (2013) phrase, been “hollowed out” at precisely the point at which aging populations have matured and where vulnerable populations have increased through recession and global change. So, we need to think about new forms of solidarity both to replace existing institutions and to indicate the basis for alternative forms of social action. Four illustrations will be made to develop this point: first, identifying forms of cooperation that bring together different generational interests; second, reconnecting to the original vision of the welfare state; third, adopting a human rights perspective in old age; and fourth, restoring meaning and dignity to the end of life.

The first issue here is how we think about new forms of cooperation that bring together rather than divide generations. Castells, Caraca, and Cardoso (2013) argue that the crisis in global capitalism is producing a new economic culture, one that expands “decommodified” modes of production. For example, he highlights the degree to which practices such as voluntary activity, cooperative community groups, and time banks have become more significant in the context of the shrinkage of monetary-based consumption. In the case of older people, examples can be cited such as University of the Third Age (U3A), first established in France in 1972 and subsequently developed across Europe and elsewhere. Here, members become teachers as well as learners, fostering a “self-help” ideal based on the knowledge that retired people are experts in many fields. In the UK, by 2011, U3A had established more than 200 groups with a membership reaching beyond 200,000. Another important area for informal education has been the development of “intergenerational learning”—that is, educational programs that link older with younger learners. Newman and Hatton-Yeo (2008) cite the example of the NUGRAN program at the University of Valencia, which creates learning experiences that cross the generations, involving older and younger adults together, with the aim of promoting greater contact, trust, and more positive attitudes between them. The program began with 71 students in 1999 and had expanded to 1,000 by 2007. Achenbaum (2005:61) provides similar examples from the United States, citing in particular the partnership between the University of North Carolina at Asheville and the North Carolina Center for Creative Retirement: “Participants take classes that they design, conduct inter-generational programs to develop leadership skills, and analyze problems in the community and at the state level.”

Another form of intergenerational solidarity recognizes the common interests of older and younger generations in protecting and improving the environment. Steinig and Butts (2010: 64) emphasize that

Older adults are concerned about the world they are leaving behind for their grandchildren and other younger generations. The young are concerned about the state of the world they will inherit. Both populations are often particularly susceptible to the same environmental health hazards—air pollution days for example. They are each at an age when they are more likely to live life in their neighborhoods and have more flexibility in their schedules than working, commuting middle generations.

Pillemer et al. (2010) argue that older people represent an important source for creating solutions to environmental problems through volunteering and civic engagement, drawing upon their own knowledge and experience (see Achenbaum 2005). Steinig and Butts (2010), discussing the U.S. experience, highlight the fact that intergenerational strategies can have a positive impact on the environment through shared sites and housing developments that bring generations together. More generally, such work has the benefit of bringing together “the collective resources of the aging and environmental constituencies to encourage senior volunteers to work together to enhance a community's environment for present and future generations” (Wright and Lund 2010:15).

A second area for promoting solidarity might come through reconnecting to the original vision of a welfare state with responsibility for promoting the well-being of all its citizens. The tendency for capitalism to convert “public services into commodities” (Navarro 1976) has been vastly accelerated over the past decade, with increasing penetration of multinational corporations into the health and social care system (Davis and Tallis 2013; Estes et al. 2013). The evidence suggests that this process has had the greatest effect on the poorest elderly, with community services for low-income groups most vulnerable to underfunding and potential closure (Hermann 2010). Leys (2010) draws a wider argument from this about the importance of what he calls “protected social spaces, which are orientated to the common welfare and which cannot be trusted to the blind power of the market.” He argues that

We have to respect and sustain areas in which communication and co-operation is not commercialised, where services do not have the character of commodities. Such protected sectors extend from the way vulnerable groups are dealt with … to social goals such as solidarity and equity and vulnerable communication structures –especially those which are based on confidence like the … worker-patient relationship. Indeed, these protected social spaces form the basis for a humane social model. (P. 25)

Developing such spaces will be especially important given the need to protect people with Alzheimer's and other forms of dementia and those with major physical disabilities, from the dangers of abusive relationships both in the community and in institutional settings. The need to embed care relationships within spaces that emphasize solidarity over market forces is an important task for gerontologists and others to address.

A third area for promoting solidarity might also come from the adoption of the type of human rights perspective developed in the later writings of Peter Townsend (2007). He highlighted the importance of measures such as the *European Convention on Human Rights* and *The Universal Declaration of Human Rights* as offering a means of challenging the “structured dependency” of older people (Townsend 1981). Use of such frameworks may become essential given the rise of care organizations operating across national borders and the drive to deregulate and privatize hitherto public services (Estes and Associates 2001; Davis and Tallis 2013). Townsend (2007:43) argued that problems relating to dependency persisted as a major issue affecting older people, with these problems set to grow in many parts of the world. At the same time, he concluded that “[h]uman rights instruments offer hope of breaking down blanket discrimination and of using resources more appropriately, and more generously, according to severity of need. But investment in human rights is not only a moral and quasi-legal salvation from things that are going depressingly wrong. Used best, human rights offer a framework of thought and planning [for] the 21^st^ Century that enables society to take a fresh, and more hopeful, direction.”

Finally, a major challenge that will require joint work across all the academic disciplines concerns thinking about the meaning of old age and especially late old age in complex global societies. There is much criticism of the professional context within which care and treatment is carried out. Much of this reflects issues about the proper resourcing for care—whether in the community or in hospitals (Davis and Tallis 2013; Estes et al. 2013). But it may also reflect the struggle of professionals and the disciplines within which they are trained to understand the distinctive character of longevity. Although late modern society demonstrates that life can be improved in many important ways, human life in general and human aging in particular pose more questions than science can answer. Here, we need to develop meaningful ways of managing situations in life that in many respects *cannot* be controlled. Conditions such as dementia pose a particular challenge in this regard. By the time someone aged 90 years or more dies, the risk of being demented is approximately 60 percent (Le Couteur et al. 2013). But late-modern cultures of aging often have difficulty acknowledging and *dignifying* this reality. Following this, Peter Whitehouse (2007) suggests that in the case of conditions such as Alzheimer's, the way forward is to rely less on the promise of genetically based therapies, more on the importance of rethinking the social narrative with which Alzheimer's is associated. He argues:

Just as all diseases (even so-called “psychological” ones) have a biology, so, too, is every disease socially constructed. We debate and discuss and then agree (or not) on the labels we use. Saying that something is socially constructed is not to say that the condition is not real or that suffering cannot result from the phenomenology. Rather, the concept of social construction offers the hope that reframing how we think about [in this example] aging-associated cognitive challenges can lead to improvements in the quality of life of those suffering today and those who will suffer in the future. … Do we remove the hope when we are more realistic about the likelihood of cure and more effective therapies? No, we create a true hope that puts the responsibility of addressing brain aging in the hands of ourselves and our communities rather than in the domain of pseudoscientific and scientistic prophecy. (P. 460)

## Conclusion

The aim of this article has been to examine the institutional changes affecting aging in the 21st century, viewing these from the standpoint of accelerated globalization and economic recession. A major issue identified in the discussion has been the tension that has emerged between longevity on the one hand and a more unequal old age on the other (Cann and Dean 2009). This must itself be linked to the weakening of institutional supports linked with old age, notably in relation to retirement and the welfare state. The stakes are now high in terms of the future of support for older people. As Gray (2010:5) has observed, “A roll-back of the state of the magnitude [planned] will leave people more exposed to the turbulence of world markets than they have been for generations. Inevitably, they will seek protection.” Yet the nature of such support will inevitably be different than that which shaped the lives of older people in the second half of the 20th century. Then, it was a relatively modest welfare state that provided a “moral narrative” for growing old; then, it was the promise of retirement that underpinned the aspirations of many—at least in industrial countries; then, it was the idea of a compact between generations that was presented as a new form of “bonding” for society. Analysis now suggests a more unequal and divided old age, with prosperity for some matched by deep poverty for others—all of this complicated by the greater spread of incomes within different cohorts and added threats introduced by global risks and insecurities (Stiglitz and Kaldor 2013).

But at the very least all of the above should be seen as a challenge to develop new forms of analysis and institutional alternatives. Society is now faced with a different type of aging underpinned by changing cultural and economic forces and responses. These are transforming the landscape around which the social construction of aging has traditionally been built. This article has identified new forms of solidarity that need to be built within the context of aging societies. Given the weakening of institutional supports for later life, redefining the basis of social solidarity between and within generations is an important task. Sociology has been slow to respond to the changes facing older people—notably those most affected by policies that have undermined institutions associated with retirement and the welfare state. Developing new ways of analyzing and responding to the challenges facing older people is likely to remain at center stage in theoretical and policy debate for some years ahead.
